# A Adiponectina Previne a Reestenose pela Inibição da Proliferação Celular em um Modelo de Enxerto Venoso em Ratos

**DOI:** 10.36660/abc.20200761

**Published:** 2021-11-22

**Authors:** Yang Zhou, Chun Dai, Bing Zhang, Jianjun Ge

**Affiliations:** 1 Anhui Medical University Anhui Provincial Hospital Department of Cardiac Surgery Hefei Anhui China Department of Cardiac Surgery, Anhui Provincial Hospital, Anhui Medical University, Hefei, Anhui - China; 2 Shandong University Cheeloo College of Medicine School of Medicine P.R. China School of Medicine, Cheeloo College of Medicine, Shandong University, P.R. – China; 3 University of Science and Technology of China Hospital of USTC Division of Life Sciences and Medicine Hefei Anhui China The First Affiliated Hospital of USTC, Division of Life Sciences and Medicine, University of Science and Technology of China, Hefei, Anhui - China

**Keywords:** Adiponectina, Proliferação de Células, Hiperplasia

## Abstract

**Fundamento:**

O enxerto de bypass na artéria coronária (CABG) continua a ser eficiente como tratamento para pacientes portadores de doença arterial coronariana; entretanto, o enxerto venoso tende a apresentar reestenose ou oclusão. A adiponectina (ADP) é uma proteína hormonal plasmática com a função de regular a proliferação celular.

**Objetivo:**

Foram utilizadas duas doses diferentes da proteína ADP em um modelo de enxerto venoso em ratos para estimular a alteração do enxerto venoso. O objetivo deste estudo foi investigar o efeito da ADP sobre a reestenose em enxerto venoso.

**Métodos:**

Veias jugulares autólogas foram implantadas como enxertos interposicionais de carótida pela técnica de anastomose de manga em ratos Sprague Dawley. A adiponectina (2,5 *μ*g e 7,5 *μ*g) foi entregue ao enxerto venoso por bypass de forma perivascular, suspensa em gel Pluronic-F127 a 30%. O grupo tratado apenas com bypass e o grupo tratado com gel veículo carregado apenas com Pluronic funcionaram como controle. Foram feitas comparações com análise de via única de variância e teste post-hoc, com p <0,05 sendo considerado significativo.

**Resultados:**

A proliferação celular (índice de PCNA) foi significativamente baixa no grupo tratado com adiponectina em comparação com o grupo de controle e o grupo tratado com o gel veículo na íntima e na adventícia dos enxertos a partir do dia 3 (p <0,01). VCAM-1 e ICAM-1 avaliados por imuno-histoquímica diminuíram significativamente em enxertos venosos tratados com adiponectina na quarta semana (p <0,01). O tratamento de enxertos venosos com gel carregado com adiponectina reduziu a espessura da íntima, da média e da adventícia, em comparação com os enxertos de controle e tratados com gel veículo no dia 28 (p <0,01).

**Conclusões:**

Este estudo oferece evidências adicionais do possível papel terapêutico da adiponectina na modulação de lesão vascular e seu reparo.

## Introdução

A doença arterial coronariana (DAC) é uma doença mundial com crescente morbidade e mortalidade.^1^ O enxerto de bypass na artéria coronária (CABG) continua a ser eficiente como tratamento para pacientes em estágio avançado. Enxertos venosos de safena oferecem o duto para bypass mais amplamente utilizado, e representam ~50% dos enxertos utilizados em CABG devido à conveniência de sua retirada, e por não serem facilmente afetados pelo fluxo de sangue coronariano concorrente.^2,3^ Apesar das vantagens do tratamento, a veia enxertada tem a tendência à reestenose ou oclusão sob a influência da pressão arterial, inflamação persistente e riscos de falha associados, com um índice de patência de 65% - 80% em 5 anos após a operação.^4–6^ Portanto, a inibição a reestenose nos enxertos venosos ainda representa um desafio.

A hiperplasia da íntima (HI) tem um papel causal na reestenose de veias enxertadas, caracterizada pelo acúmulo excessivo de células musculares lisas vasculares (CMLV) levando a distúrbios oclusivos.^6–8^ Além disso, há cada vez mais dados que indicam que a adventícia também pode estar relacionada à remodelação das veias enxertadas.^9–11^ Depois da lesão, os fibroblastos da adventícia se ativam e proliferam, seguidos pela biossíntese da adventícia, e liberam uma variedade de citocinas para promover a proliferação de CMLV. Todo o processo resulta em HI e reestenose.^12–15^ Comprovou-se que muitas citocinas estão envolvidas na proliferação das CMLV, entre as quais VCAM-1 e ICAM-1, que podem promover a adesão das células e a formação da lesão aterosclerótica.^16,17^

A adiponectina (ADP), uma proteína hormonal plasmática especificamente biossintetizada por adipócitos, pode exercer seu efeito ligando ao receptor de adiponectina nos fibroblastos.^18,19^ Além disso a ADP pode ser usada para atenuar a disfunção endotelial.^20^ Pesquisas recentes demonstram que a ADP desempenha papéis importantes na supressão da proliferação celular após o dano aos vasos, bem como diminui as citocinas que promovem a proliferação celular.^21,22^

Considerando esses achados, levantou-se a hipótese de que a ADP pode evitar a reestenose do enxerto venoso pela inibição da proliferação celular. A ADP foi aplicada à superfície externa de enxertos venosos com duas concentrações diferentes para investigar o efeito da ADP em reestenoses de enxertos venosos. Os resultados demonstraram que a ADP reduziu significativamente as espessuras de íntima, média e adventícia no enxerto venoso. Enquanto isso, a proliferação celular e a expressão das citocinas (VCAM-1 e ICAM-1) também diminuíram. Esses resultados demonstraram primeiramente o papel da ADP na prevenção da reestenose de enxertos venosos e esclareceram o possível mecanismo.

## Métodos

Este estudo recebeu aprovação ética para experimentos com animais do Comitê de uso e cuidado com animais do Primeiro Hospital Afiliado à Universidade de Ciência e Tecnologia da China. Ratos Sprague Dawley (SD) (machos e fêmeas, com idade 10-12 semanas, peso corporal de 275-325 g e N=72) foram comprados do Centro de Pesquisa Animal do Laboratório de Anhui e foram distribuídos aleatoriamente entre grupos de tratamento com ADP (grupos de dose baixa e alta), grupo de tratamento com gel veículo (gel Pluronic-F127) e grupo de controle (apenas o bypass). De acordo com pesquisas anteriores, 72 ratos foram divididos em 4 grupos, que foram posteriormente divididos em 12 grupos em três momentos do estudo diferentes.^23^ (n = 6 por grupo).

### Construtos para entrega perivascular de fármacos

A abordagem de entrega de fármacos à adventícia utilizou o gel Pluronic F127 como veículo. Um total de 2,5 *μ*g de adiponectina (Abcam) foi dissolvido em 25 *μ*l de água destilado e ressuspenso em 300 *μ*l de gel Pluronic-F127 a 30% para entrega nos enxertos venosos após a interposição da carótida, no grupo de tratamento com dose baixa (grupo L-ADP), enquanto 7,5 *μ*g de adiponectina foram usados para revestir os enxertos venosos no grupo de tratamento com alta dose de adiponectina (grupo H-ADP). Uma quantidade igual de gel Pluronic-F127 foi aplicada ao grupo com tratamento por gel veículo (grupo VG). O gel Pluronic-F127 só foi retirado do ambiente refrigerado a 4 °C no momento da aplicação, para que mantivesse sua forma líquida.

### Modelo de enxerto venoso autólogo em ratos

Ratos SD foram anestesiados com hidrato cloral a 10% (300 mg/kg) e receberam heparina (200 U/kg) via injeção venosa caudal. A veia jugular esquerda foi retirada para utilização como enxerto interposicional de carótida. O bypass foi realizado pelo modelo de anastomose de manga, conforme descrito anteriormente.^24^ Especificamente, foram cortadas mangas de 2 mm de agulhas arteriais vermelhas 20G (BD Company). A artéria carótida foi isolada até as ramificações. Foram colocadas linhas de tração para sutura e clipes de hemostasia para bloquear o fluxo sanguíneo nas extremidades proximal e distal da artéria antes de serem seccionadas na metade. As duas extremidades das artérias foram puxadas por dentro da manga. Em seguida, as artérias foram evertidas e presas à manga por sutura de seda 6-0. Após a arteriotomia na artéria carótida comum esquerda (ipsilateral), as mangas foram inseridas e presas à artéria com sutura de seda 6-0. A artéria foi seccionada para permitir a extensão longitudinal do enxerto venoso interposicional. Foram aplicados tratamentos à superfície externa do enxerto após a retirada dos clipes. Os dois grupos de tratamento com a adiponectina foram submetidos à aplicação perivascular de 300 *μ*L de gel de ADP, o grupo de tratamento com gel veículo, com 300 *μ*L de gel Pluronic-F127, e o grupo de bypass apenas não recebeu gel. Além disso, o gel foi mantido resfriado até a aplicação perivascular e, em seguida, foi permitido que se solidificasse à temperatura corporal, como descrito anteriormente.^25^

### Coleta de enxertos implantados

Os ratos foram sacrificados no 3°, 14° e 28° dias após o transplante de bypass, 6 ratos por vez. Após a patência dos enxertos venosos ter sido testada por ultrassonografia por Doppler, foram coletados corpos de prova de enxertos venosos, que foram divididos em 2 seções. Um dos segmentos foi imediatamente afixado após a perfusão com solução salina heparinizada, seguida de formaldeído a 4% para hematoxilina e eosina (H&E), coloração de tricrômio de Masson e análise de ensaios imuno-histoquímicos, enquanto o outro foi colocado a −80 °C para realização do ensaio de Western blot. Os animais foram sacrificados por overdose de pentobarbital sódico.

### Análise morfométrica

Após a fixação em formaldeído a 4% e o processamento em álcool 70%, os corpos de prova coletados no 3°, 14° e 28° dias foram embebidos em parafina; foram retiradas seções de 3 *μ*m ao longo do enxerto; exceto pelas regiões imediatamente adjacentes às mangas da anastomose. As seções foram coloridas com hematoxilina e com o kit de Masson (Shanghai Gefan Biological, China), e as seções coloridas foram observadas em um microscópio biológico Leica-DM2000 (Leica, Hefei, China). Pelo menos cinco seções dos enxertos de bypass espaçadas simetricamente foram analisadas para cada corpo de prova. Medidas morfométricas padrão foram registradas, incluindo a espessura íntima, a espessura média, e a espessura adventícia. Em seguida, esses dados foram calculados pelo software ImageJ (National Institutes of Health, Bethesda, MD, EUA).

### Análise imuno-histoquímica

Tecidos embebidos em parafina (enxertos no 3°, 14° e 28° dias) foram processados, e foram retiradas seções de 3 *μ*m ao longo do enxerto; exceto pelas regiões imediatamente adjacentes às mangas da anastomose. Depois da desparafinização, o antígeno foi recuperado com tampão citrato por 20 minutos em temperatura e pressão altas, depois desativado com peroxidase endógena H_2_O_2_ a 3% for 20 minutos, e bloqueio de peroxidase endógena com albumina sérica bovina a 5% por 10 minutos. As seções foram tratadas durante a noite a 4°C com anticorpo monoclonal anti-PCNA (D3H8P; Cell Signaling), anticorpo anti-VCAM1 (ab134047; Abcam, diluição 1:500), e anticorpo anti-ICAM1 (ab119871; Abcam, diluição 1:200). Três seções separadas por enxerto foram analisadas em relação a todos os marcadores. Os cortes foram lavados várias vezes. Foram adicionadas aos cortes a solução funcional de anticorpo secundário biotinilado, e de solução funcional de estreptavidina conjugada com horseradish peroxidase. Os cortes foram lavados em PBS (pH 7.2) por 5 minutos. Foi utilizada a diaminobenzidina tetrahidrocloreto (DAB) para a visualização, e a hematoxilina foi utilizada para contracoloração. Por último, os cortes foram desidratados, e em seguida foram montadas e seladas. Todas as imagens foram capturadas por aquisição de sinal de imagem e sistema de análise (Leica-DM2000, Hefei, China). O índice de PCNA foi utilizado para descrever o nível de expressão de PCNA (índice de PCNA = número de células com PCNA positivo/número total de células). A densidade ótica média foi utilizada para descrever o padrão de expressões de VCAM-1 e ICAM-1 (densidade ótica média = densidade ótica integral/área).

### Análise de expressão de proteína VCAM-1 e PCNA pelo ensaio de Western blot

As proteínas totais foram isoladas das veias enxertadas. Após a eletroforese e a eletrotransferência, as membranas foram bloqueadas com leite desnatado a 5%, e foram incubadas a 4°C com anticorpos primários durante a noite (com anticorpo monoclonal anti-PCNA D3H8P; Cell Signaling, diluição 1:1000; anticorpo anti-VCAM1 ab134047; Abcam, diluição 1:3000; anticorpo anti-β-tubulina GB11017; Servicebio, diluição 1:2000). Depois de serem incubadas com anticorpos secundários (IgG de cabra anti-IgG de camundongo HRP SE131; Solarbio, diluição 1:3000), as membranas foram lavadas com TBST. Em seguida, as membranas foram expostas no filme de PVDF e a solução de reagente ECL (PE0010, Solarbio) foi adicionada. Faixas imunorreativas separadas foram processadas pelo software Odyssey v1.2. Valores cinza foram medidos com base na referência interna da β-Tubulina.

### Análise estatística

Os dados foram apresentados como média ± desvio padrão (DP) e foram processados pelo software SPSS v.21.0 (Chicago, IL, EUA). Como os dados seguiram a distribuição normal verificada pelo teste Kolmogorov–Smirnov, as comparações entre vários grupos foram analisadas por análise de variância de via única (ANOVA). As comparações entre dois grupos foram analisadas pelo teste de diferença mínima significativa de Fisher (LSD). Um p-valor <0.05 foi considerado estatisticamente significativo.

## Resultados

**Figura 1:**
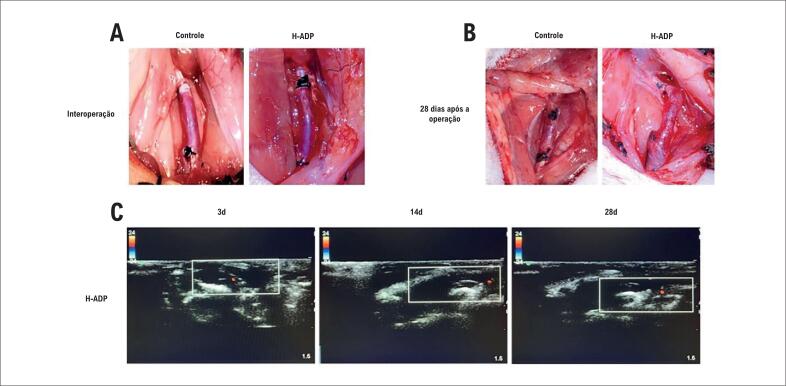
Fotografias intraoperatórias e pós-operatórias e fotografias do ultrassom por Doppler. (A) a. Fotografias intraoperatórias dos grupos de controle e alta dose de adiponectina; (B) fotografia pós-operatória na quarta semana dos grupos de controle e de alta dose de adiponectina; (C) fotografias do ultrassom por Doppler no dia da eutanásia dos grupos de controle e alta dose adiponectina.

O método de operação descrito anteriormente foi usado para estabelecer o modelo de bypass autólogo em veia jugular em ratos. Após o procedimento, as veias jugulares transplantadas foram totalmente preenchidas, e os vasos sanguíneos tinham bons batimentos. A entrega perivascular local do gel Pluronic-F 127 cobriu uniformemente a superfície do vaso sanguíneo. A incisão foi fechada após a solidificação do adesivo. Os status de atividade e cura da incisão nos ratos foram verificados diariamente após a operação. Foi realizado ultrassom por Doppler no dia da eutanásia, que demonstrou que o sangue da veia jugular transplantada não apresentava uma clara oclusão. No grupo de controle de 28 dias, os enxertos venosos estavam espessados e ligeiramente rígidos, e a adesão dos tecidos adjacentes estava relativamente óbvia. Correspondentemente, no grupo de alta dose de adiponectina, as veias não se expandiram significativamente, e os tecidos adjacentes poderiam ser separados com facilidade (Figura 1).

A adiponectina atenua a hiperplasia de íntima, média e adventícia em enxerto venoso

**Figura 2:**
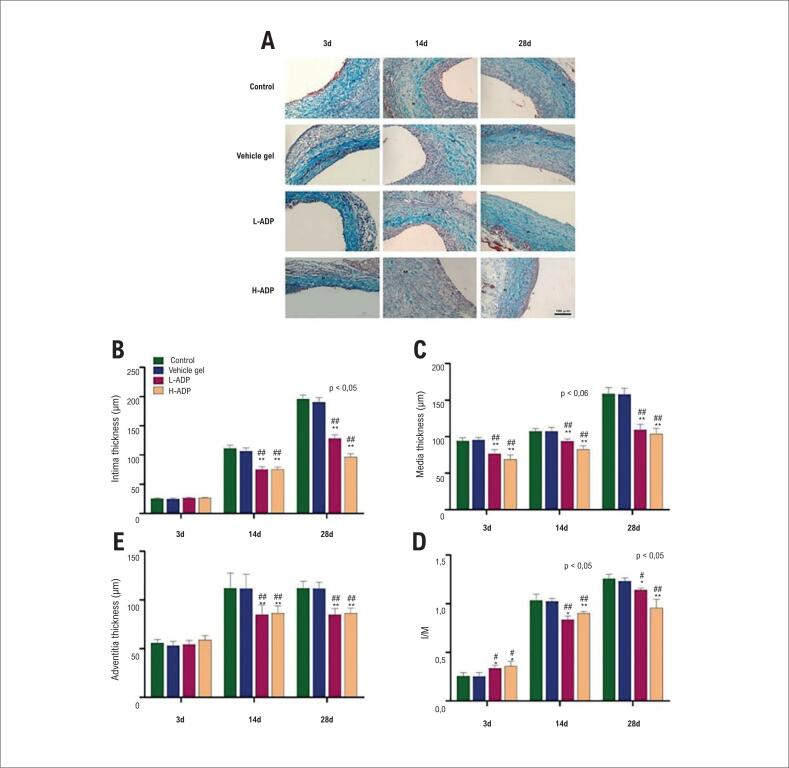
A entrega perivascular local de adiponectina atenua a hiperplasia de íntima, média e adventícia em enxerto venoso em ratos. (A) Microfotografias de seções de veias enxertadas com coloração de Masson (200× ampliação, barra: 100 *μ*m). (B) Mudança da espessura neoíntima; (C) Mudança da espessura da média; (D) Mudança da espessura da adventícia; (E) relação íntima-média (I/M). Os resultados são apresentados como média + ± DP. *p <0,05 versus grupo de controle, **p <0,01 versus grupo de controle. #p <0,05 versus grupo de gel veículo, ##p <0,01 versus grupo de gel veículo. I: íntima; M: média.

Análises morfométricas de enxertos explantados após o bypass (Figura 2A) apresentaram redução da espessura íntima, média e adventícia após a entrega perivascular da adiponectina. A quantificação da espessura íntima, média e adventícia foi realizada por, pelo menos, cinco seções igualmente espaçadas ao longo da veia não anastomótica em cada enxerto do bypass. Na visualização dos três momentos de análise, dia 3, dia 14 e dia 28, não houve diferença significativa entre a espessura da íntima, da média e da adventícia entre o grupo de controle e o grupo de gel veículo, o que significou que a aplicação do gel Pluronic F-127 como carga de gel não afetou a fisiopatologia dos enxertos venosos. Especificamente, a espessura da íntima do enxerto venoso no grupo H-ADP diminuiu significativamente no dia 14 e no dia 28 em comparação com o grupo de controle e o grupo de gel veículo. De forma semelhante, a espessura da íntima no grupo L-ADP evidentemente reduziu mais, em comparação com o grupo de controle e o grupo de gel veículo no dia 14 e no dia 28 (Figura 2B, Tabela 1).

**Tabela 1:** Espessura da íntima, média e adventícia nos grupos de controle, de gel veículo (Pluronic-F127), L-ADP (dose baixa de ADP) e H-ADP (dose alta de ADP).

Grupos	Horário (dia)	Controle N=6	Gel veículo N=6	L-ADP N=6	H-ADP N=6	Análise
Espessura da íntima (μm)	3	25,47±1,02	25,10±1,53	26,55±1,05	26,46±0,71	ANOVA
	14	111,69±5,31	106,62±5,57	75,12±4,94** ##	75,46±3,59** ##	ANOVA
	28	196,09±6,78	190,44±7,68	128,30±6,01** ##	96,96±5,45** ##	ANOVA
Espessura da média (μm)	3	94,00±4,19	95,47±3,38	76,93±5,30** ##	69,03±5,94** ##	ANOVA
	14	107,19±3,66	107,41±5,48	93,86±3,17** ##	82,83±4,13** ##	ANOVA
	28	158,83±8,39	157,75±8,75	109,67±7,16** ##	104,02±7,12** ##	ANOVA
Espessura da adventícia (μm)	3	56,09±3,42	53,03±4,59	54,51±3,95	59,21±4,28	ANOVA
	14	112,03±7,08	111,87±6,35	85,04±6,35** ##	86,52±5,20** ##	ANOVA
	28	152,31±3,55	154,49±6,55	95,70±6,05** ##	90,15±4,87** ##	ANOVA

Os valores das espessuras são apresentados como média ± DP (=6).

* p <0,05 versus grupo de controle,

** p <0,01 versus grupo de controle.

# p <0,05 versus grupo de gel veículo,

## p <0,01 versus grupo de gel veículo.

Comparada à hiperplasia da íntima, a hiperplasia da média nas veias enxertadas ocorreu mais cedo; o espessamento da média no grupo de controle e no grupo de gel veículo, no dia 3 era mais óbvia que nos grupos L-ADP e H-ADP. Similarmente, a espessura do tecido da média diminuiu nos grupos com alta dose de adiponectina comparados aos grupos de controle e de gel veículo no dia 14 e no dia 28. A mesma tendência também existiu no grupo de baixa dose de adiponectina no dia 14 e no dia 28 (Figura 2C, Tabela 1).

As relações íntima-média nos grupos L-ADP e H-ADP foram mais altas em comparação com grupo de controle e o de gel veículo no dia 3. Posteriormente, a relação íntima-média era notavelmente mais baixa no grupo H-ADP no dia 14 e no dia 28 em comparação com o grupo de controle e o grupo de gel veículo. Dados do grupo L-ADP revelaram uma tendência similar no dia 14 e no dia 28 versus o grupo de controle e de gel veículo (Figura 2E).

Além disso, identificou-se que a aplicação de adiponectina também pode inibir a hiperplasia da adventícia de veias enxertadas em ambos os grupos com doses diferentes. A espessura da adventícia foi atenuada pela alta dose de adiponectina comparada aos grupos de controle e de gel veículo no dia 3, no dia 14 e no dia 28. A mesma tendência também existiu no grupo de baixa dose de adiponectina comparada aos grupos de controle e de gel veículo no dia 3, no dia 14 e no dia 28 (Figura 2D, Tabela 1).

### A adiponectina diminui a proliferação celular após o enxerto venoso

**Figura 3:**
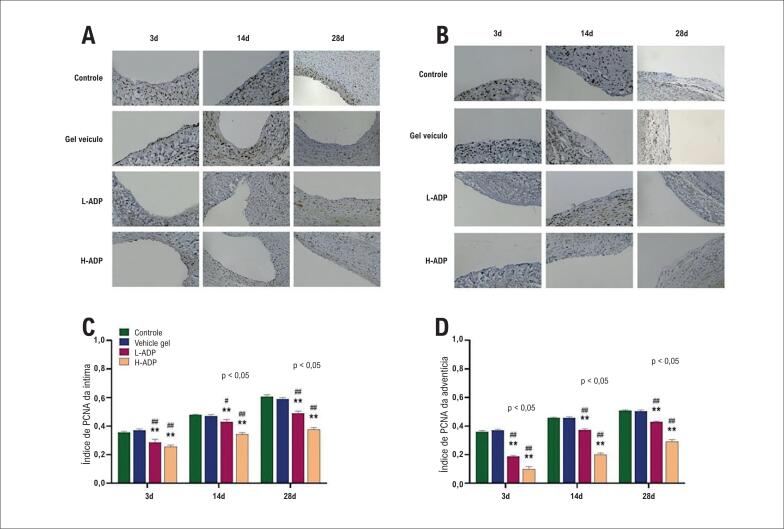
A adiponectina diminui a proliferação celular. (A) Índice de PCNA da íntima determinado por imuno-histoquímica (200×ampliação); (B) índice de PCNA da adventícia determinado por imuno-histoquímica (200×magnificação); (C) índice de PCNA da íntima; (D) índice de PCNA da adventícia. Os dados representam média + ± DP. *p <0,05 versus grupo de controle, **p <0,01 versus grupo de controle. #p <0,05 versus grupo de gel veículo, ##p <0,01 versus grupo de gel veículo.

**Figura 4:**
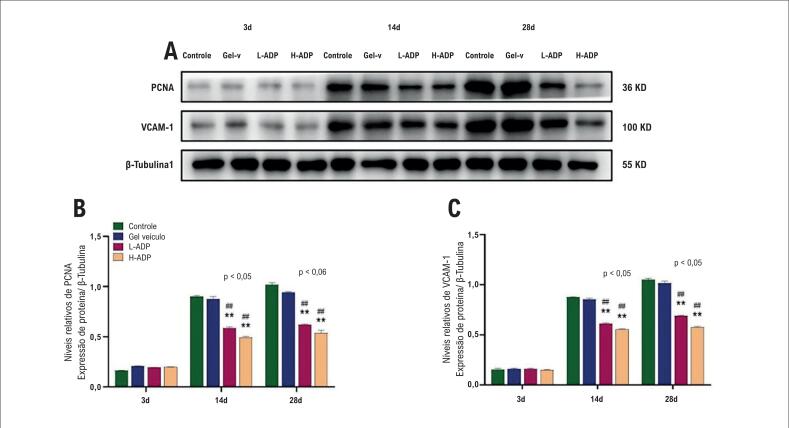
Inibição da expressão de PCNA e VCAM-1 pela entrega de adiponectina. (A) Expressões de PCNA e VCAM-1 em todos os grupos em três momentos no tempo após a operação; (B) Expressão de PCNA determinada por Western blot; (C) Expressão de VCAM-1 determinada por Western blot. Os dados representam média + ± DP. *p <0,05 versus grupo de controle, **p <0,01 versus grupo de controle. #p <0,05 versus grupo de gel veículo, ##p <0,01 versus grupo de gel veículo.

A proliferação celular foi quantificada pelo índice de PCNA da íntima e da adventícia (número total de células com PCNA positivo dividido pelo número total de células nucleadas) em 3 momentos no tempo após o bypass (Figuras 3A, 3B). A proliferação celular entre o grupo de controle e o grupo de gel veículo na íntima e na adventícia foi similar nos três momentos no tempo. Veias tratadas com alta dose de adiponectina demonstraram proliferação mínima na íntima (26%) e na adventícia (10%) três dias após o bypass em relação aos grupos de controle e de gel veículo. Em comparação com os grupos de controle (íntima: 36%; adventícia: 36%) e de gel veículo (íntima: 37%; adventícia: 37%), a proliferação diminuiu no grupo L-ADP na íntima (29%) e na adventícia (19%) no terceiro dia. Além disso, a supressão da proliferação do grupo H-ADP também foi mais evidente na adventícia do que no grupo L-ADP no dia 3. A proliferação foi pronunciada em enxertos venosos no dia 14 (íntima: 48%; adventícia: 46%) e no dia 28 (íntima: 61%; adventícia: 51%) no grupo de controle, mas também no dia 14 (íntima, 47%; adventícia, 46%) e dia 28 (íntima, 59%; adventícia, 50%) no grupo de gel veículo. Aplicação perivascular de adiponectina com alta dose apresentou 34% de proliferação na íntima e 20% na adventícia no dia 14 quando comparado com o grupo de controle e o grupo de gel veículo. A proliferação no grupo L-ADP era visivelmente mais baixa na íntima (43%) e na adventícia (37%) no dia 14 em comparação aos grupos de controle e de gel veículo. As diferenças também podem ser encontradas na íntima e na adventícia entre os dois grupos com doses diferentes no dia 14. A inibição da proliferação também foi evidente no dia 28 no grupo L-ADP, 49% de proliferação da íntima e 43% da adventícia, o que ilustra diferenças significativas em relação aos grupos de controle e de gel veículo. A expressão do PCNA nos grupos de H-ADP apresentou uma tendência similar na íntima (38%) e na adventícia (29%) no dia 28. A comparação entre dois grupos de adiponectina diferentes também demonstrou diferenças notáveis na íntima e na adventícia no dia 28 (Figuras 3C, 3D). A expressão do PCNA determinada pelo teste de Western Blot também demonstrou os mesmos efeitos da adiponectina (Figuras 4A, 4B).

### A adiponectina diminui as expressões de VCAM-1 e ICAM-1 após o enxerto venoso

**Figura 5:**
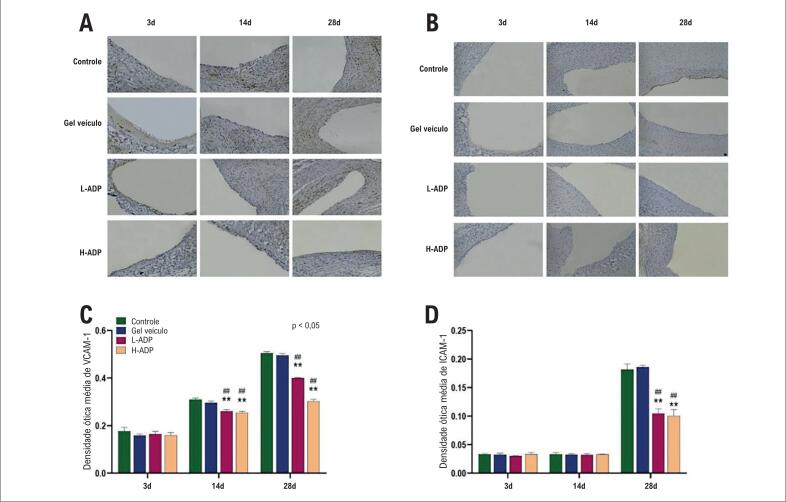
A adiponectina diminui expressões de citocina após o enxerto venoso. (A) Expressão de VCAM-1 determinada por imuno-histoquímica (200×ampliação); (B) Expressão de ICAM-1 determinada por imuno-histoquímica (200×ampliação); (C) Expressão de VCAM-1; (D) Expressão de ICAM-1. Os dados representam média + ± DP. *p <0,05 versus grupo de controle, **p <0,01 versus grupo de controle. #p <0,05 versus grupo de gel veículo, ##p <0,01 versus grupo de gel veículo.

VCAM-1 foi expressado no estágio inicial, mas não houve diferenças entre os grupos no dia 3. A diferença não foi observada entre os grupos de controle e de gel veículo. A expressão de VCAM-1 no grupo de tratamento com alta dose de adiponectina foi significativamente suprimida em relação aos grupos de controle ou de gel veículo no dia 14 (0,25 versus 0,31, 0,30) e no dia 28 (0,30 versus 0,50, 0,50). A expressão de VCAM-1 no grupo L-ADP também foi inibida em relação aos grupos de controle ou de gel veículo no dia 14 (0,26) e no dia 28 (0,40). *Além disso*, a diferença entre os grupos H-ADP e L-ADP também foi marcada no dia 28 (Figura 5A, Figura 5C). Os resultados determinados pelo teste Western Blot também ilustraram uma tendência similar (Figura 4A, Figura 4C).

A expressão de ICAM-1 analisada pelo ImageJ raramente foi observada nos estágios iniciais. As expressões de ICAM-1 não mostraram diferença significativa no grupo H-ADP (0,10 versus 0,18 no grupo de controle, 0,19 no grupo de gel veículo) e no grupo L-ADP (0,10 versus grupos de controle e de gel veículo) até o dia 28 (Figura 5B). Nenhuma diferença foi observada entre os dois grupos com doses diferentes de adiponectina nos três momentos no tempo.

## Discussão

Apesar dos avanços nas terapias de DAC, o CABG continua sendo o principal tratamento cirúrgico, e, portanto, a reestenose continua a afligir a patência de enxertos venosos.^26^ Muitos experimentos tentaram entender melhor os mecanismos de falha do enxerto venoso e identificar novas abordagens para reduzir a lesão ao vaso e melhorar a cura.^27–29^ Este estudo demonstrou que a adiponectina entregue à superfície externa dos enxertos venosos em ratos no momento do implante por gel Pluronic-F não só reduziu a hiperplasia íntima no dia 28 em comparação com o grupo de controle em que só foi realizado o bypass, mas também pode diminuir a hiperplasia da média e da adventícia. Os resultados deste estudo sugerem que a atividade antiproliferativa e a expressão de citocinas (VCAM-1, ICAM-1) na parede do enxerto provavelmente devem estar envolvidas nesse efeito. Esses resultados demonstram o papel da ADP na prevenção da reestenose de enxertos venosos e esclarecem o possível mecanismo.

O processo complexo de remodelação de vasos resulta em reestenose dos enxertos venosos, mas o mecanismo ainda não está claro. Estudos demonstraram que o processo de reestenose de enxertos venosos inclui trombose, hiperplasia da íntima, formação da neoíntima, e aterosclerose.^3^ Depois do transplante dos vasos, a proliferação e a migração celular do músculo liso vascular foram afetadas pelas alterações na disfunção das células endoteliais, inflamação, hemodinâmica, entre outros fatores.^11^ Além disso, a adventícia do vaso sanguíneo também é danificada. Os fibroblastos ativados da adventícia se transformam em miofibroblastos, e se proliferam a migram para a média e íntima, onde sintetizam e liberam uma série de citocinas que promovem a proliferação e a migração celular.^30–32^ Estudos já revelaram que a adiponectina pode ser ligada a receptores de adiponectina expressos em fibroblastos da adventícia e desempenhar uma importante função. Além disso, muitos estudos relataram que a adiponectina tem efeitos anti-inflamatórios e anti-ateroscleróticos no sistema cardiovascular.^33^ Entretanto, os efeitos da ADP na prevenção da reestenose permanecem amplamente desconhecidos. Neste estudo, primeiramente demonstrou-se a possível eficácia da ADP na inibição de hiperplasia em enxertos venosos.

Neste estudo, a adiponectina foi aplicada ao modelo de enxerto venoso autólogo em ratos para estimular o processo fisiopatológico de enxertos venosos após CABG. Observou-se que a espessura da íntima nos grupos tratados com adiponectina era significativamente mais baixa que nos grupos tratados apenas com bypass e gel veículo, indicando que a adiponectina tem um efeito na inibição da hiperplasia da íntima. Além disso, a dose mais alta de adiponectina foi mais eficiente neste estudo. É interessante notar que o índice de PCNA da íntima e da adventícia também diminuiu nos grupos tratados com adiponectina, demonstrando que a adiponectina pode ter um efeito inibitório na proliferação celular na íntima e na adventícia de veias enxertadas. Este estudo apresenta apenas uma visão limitada dos mecanismos pelos quais a ADP reduziu a íntima do enxerto venoso neste modelo. Entretanto, ao comparar estes dados com os dados obtidos no contexto de estudos anteriores, concluiu-se que a adiponectina conseguia não só inibir a hiperplasia da íntima, mas também inibir a proliferação de miofibroblastos e limitar sua migração para a íntima e a média.^34,35^

Entretanto, o mecanismo específico de interação entre a adiponectina e os receptores ainda exige estudos posteriores. A farmacocinética de entrega perivascular de adiponectina também precisa ser mais bem definida em estudos futuros. Além disso, pesquisadores demonstraram que a trombose desempenhe um papel central na fase inicial das falhas de enxertos venosos. A veia é submetida a um período de lesão de isquemia e reperfusão, levando a uma redução na sintase endotelial do óxido nítrico (eNOS), expressando danos às células endoteliais e células musculares lisas vasculares (CMLV) e liberação de vários mediadores protrombóticos. Além disso, os processos de remodelação de enxerto venoso se iniciam dias após a coleta e o enxerto, levando à formação da hiperplasia da íntima.^25^ Considera-se que a ADP possa exercer um efeito nos estágios iniciais e intermediários. Este estudo apresenta informações apenas sobre os efeitos em curto prazo. Pesquisas posteriores precisam ser realizadas para que se entenda o mecanismo específico no curto prazo e para saber como a ADP influencia o resultado no longo prazo.

Depois de o endotélio ser lesionado, um processo fisiopatológico inicial de reestenose, as moléculas de adesão ICAM-1 e VCAM-1 são liberadas, o que pode levar à adesão e à infiltração de monócitos/macrófagos que diferenciam e fagocitam uma grande quantidade de ox-LDL, e, em seguida, formam células espumosas. Nestes experimentos, detectou-se a expressão de VCAM-1 e ICAM-1 por imuno-histoquímica, e identificou-se que os valores de VCAM-1 e ICAM-1 nos grupos tratados por adiponectina eram marcadamente mais baixos do que os do grupo tratado por gel veículo e do grupo tratado apenas por bypass, o que elucidou como a aplicação da adiponectina pode reduzir a expressão de VCAM-1 e ICAM-1 para promover a disfunção do endotélio.

## Conclusão

No presente estudo, confirmou-se que a entrega perivascular local de adiponectina atenua a hiperplasia de enxertos venosos em um modelo de bypass de carótida em ratos. O efeito parece ser mediado tanto pela diminuição da proliferação celular quanto pela diminuição da expressão de VCAM-1 e ICAM-1 dentro do enxerto. Embora os efeitos de curto prazo da adiponectina pareçam promissores, os efeitos de longo prazo e a significância clínica da adiponectina no CABG precisa ser estudado no futuro. Espera-se que estes estudos, em algum momento, se traduzam em aplicação clínica na prevenção de reestenose e falha nos enxertos em bypass.
