# Supplementary material for: A Adiponectina Previne a Reestenose pela Inibição da Proliferação Celular em um Modelo de Enxerto Venoso em Ratos
Source: Arq Bras Cardiol. 2021 Nov 22;117(6):1179–88. [Article in Portuguese] doi: 10.36660/abc.20200761 (PMC8757157; doi:10.36660/abc.20200761)
Supplement: Translation

# Translation: Adiponectin Prevents Restenosis Through Inhibiting Cell Proliferation in a Rat Vein Graft Model

**Authors:** Yang Zhou, Chun Dai, Bing Zhang, Jianjun Ge

**Affiliations:** 1 Hefei Anhui China Department of Cardiac Surgery, Anhui Provincial Hospital, Anhui Medical University, Hefei, Anhui - China; 2 P.R. China School of Medicine, Cheeloo College of Medicine, Shandong University, P.R. – China; 3 Hefei Anhui China The First Affiliated Hospital of USTC, Division of Life Sciences and Medicine, University of Science and Technology of China, Hefei, Anhui - China

**Keywords:** Adiponectin, Cell Proliferation, Hyperplasia

## Abstract

**Background:**

Coronary artery bypass grafting (CABG) continues to be an effective therapy for coronary artery disease patients, but the vein graft is prone to restenosis or occlude. Adiponectin (ADP) is a plasma hormone protein with the function of regulating cell proliferation.

**Objective:**

This study used two different doses of ADP protein in a rat vein graft model to stimulate vein graft change. The aim of our study was to investigate the effect of ADP on vein graft restenosis.

**Methods:**

Autologous jugular veins were implanted as carotid interposition grafts through the anastomotic cuff technique in Sprague Dawley rats. Adiponectin (2.5 *μ*g and 7.5 *μ*g) was delivered to the vein bypass grafts in a perivascular fashion, suspended in a 30% Pluronic-F127 gel. No treatment (bypass only) and vehicle loaded Pluronic gel served as controls. Comparisons were made with one-way analysis of variance and a post-hoc test, with p < 0.05 considered significant.

**Results:**

Cell proliferation (PCNA index) was significantly low in adiponectin-treated versus control and vehicle-gel-treated grafts, both in intima and adventitia, as of day 3 (p < 0.01). VCAM-1 and ICAM-1 evaluated by immunohistochemistry significantly down-regulated in the adiponectin-treated vein grafts in the fourth week (p <0.01). Treatment of vein grafts with adiponectin-loaded gels reduced intimal, media, and adventitia thickness when compared with the control and vehicle-gel-treated vein grafts at day 28 (p < 0.01).

**Conclusions:**

Our studies provide further support for the potential therapeutic role of adiponectin in modulating vascular injury and repair.

## Introduction

Coronary artery disease (CAD) is a worldwide disease with an increasing morbidity and mortality.^1^ Coronary artery bypass grafting (CABG) continues to be an therapy for advanced stage patients. Saphenous vein grafts provide the most widely used bypass conduit, which make up ~50% of grafts used in CABG for its convenience of harvesting and manipulating, its sufficient length, and its character of not easily being affected by competitive coronary blood flow.^2,3^ Despite the advantages in treatments, the grafted vein is prone to restenosis or occlude under the influence of arterial blood pressure, persistent inflammation and associated risks of failure, with a patency rate of 65% - 80% in 5 years after operation.^4–6^ Therefore, how to inhibit restenosis in the vein grafts represents a challenge.

Intimal hyperplasia (IH) plays a causal role in grafted vein restenosis, characterized by an excessive accumulation of vascular smooth muscle cells (VSMCs) leading to occlusive disorders.^6–8^ In addition, increasing data indicate that the adventitia may also be related to the remodeling of the grafted veins.^9–11^ After being injured, adventitial fibroblasts activate and proliferate, followed by adventitia biosynthesizing, and release a variety of cytokines to promote the proliferation of VSMCs. The entire process results in IH and restenosis.^12–15^ Many cytokines have proven to be involved in the proliferation of VSMCs, among which VCAM-1 and ICAM-1 can promote cell adhesion and atherosclerotic lesion formation.^16,17^

Adiponectin (ADP), a plasma hormone protein specifically biosynthesized by adipocytes, can exert an effect by binding to adiponectin receptors in fibroblasts.^18,19^ ADP can also be used to attenuate endothelial dysfunction.^20^ Recent studies have shown that ADP plays important roles in suppressing cell proliferation after damage to the vessel, and decreases cytokines, which also promote cell proliferation.^21,22^

Considering these findings, we hypothesize that ADP can prevent vein graft restenosis by inhibiting cell proliferation. ADP was delivered to the external surface of the graft veins with two different concentrations in order to investigate the effect of ADP on vein graft restenosis. The results showed that the ADP significantly decreased intimal, medial, and adventitial thickness of the vein graft. Meanwhile, cell proliferation and cytokine expression (VCAM-1, ICAM-1) also decreased. These results primarily demonstrated the role of ADP in preventing vein graft restenosis and highlighted the potential mechanism.

## Methods

Ethical approval for animal experiments was accepted from the Animal Care and Use Committee of the First Affiliated Hospital to the University of Science and Technology of China. Sprague Dawley (SD) rats (male and female, aged 10-12 weeks, body weight of 275-325 g, and N=72) were purchased from the Anhui Lab Animal Research Center and were randomly assigned to the ADP treatment groups (low and high dose groups), vehicle gel treatment group (pluronic-F127 gel), and control group (bypass only). According to previous research, 72 rats were divided into 4 groups, which were then divided into 12 groups at three different points of time^23^ (n = 6 per group).

### Perivascular drug delivery constructs

The adventitial drug delivery approach used Pluronic F127 gel as a vehicle gel. A total of 2.5*μ*g adiponectin (Abcam) was dissolved in 25 *μ*l of distilled water and resuspended in 300 *μ*l of 30% pluronic-F127 gel for delivery to vein grafts after carotid interposition in a low dose adiponectin treatment group (L-ADP group), while 7.5*μ*g adiponectin was used to coat the graft veins in a high dose adiponectin treatment group (H-ADP group). An equal amount of pluronic-F127 gel was applied to the vehicle gel treatment group (VG group). Only upon application were the pluronic-F 127 gels taken out of a 4°C environment, which seeks to maintain its liquid form.

### Rat autologous vein graft model

SD rats were anesthetized with a 10% chloral hydrate (300 mg/kg) and received heparin (200 U/kg) via caudal vein injection. The left jugular vein was harvested for use as a carotid interposition graft. The bypass was performed by applying the anastomotic cuff model, as previously described.^24^ Specifically, 2-mm cuffs were cut from a 20G red arterial puncture needle (BD Company). The carotid artery was isolated up to the branches. The proximal and distal ends of the artery were then placed under suture traction lines and hemoclips to block blood flow before being cut in middle. Both ends of the arteries were then pulled through the cuff. The arteries were then everted and secured to the cuff with 6–0 silk suture. Following an arteriotomy in the left (ipsilateral) common carotid artery, the cuffs were inserted into and secured to the artery with a 6–0 silk suture. The artery was then divided to enable a longitudinal extension of the vein interposition graft. Treatments were applied to the external surface of the graft after unclamping. Both adiponectin treatment groups underwent a perivascular application of 300 *μ*L of ADP gel, the vehicle gel treatment group with 300 *μ*l of pluronic-F127 gel, and the bypass only group with no gel. Additionally, gels were kept on ice until perivascular application and subsequently allowed to solidify at body temperature, as previously described.^25^

### Harvest of implanted grafts

Rats were euthanized on the 3^rd^, 14^th^, and 28^th^ days after bypass transplantation respectively, 6 rats each time. Following vein grafts patency tested by Doppler ultrasonography, vein graft specimens were harvested and cut into 2 sections. One segment was immediately fixed after perfusion with heparinized saline, followed by 4% formaldehyde for hematoxylin and eosin (H&E), Masson staining and immunohistochemistry assay analyses, while the other was placed at -80°C for Western blot analysis. Animals were sacrificed by sodium pentobarbital overdose.

### Morphometric analysis

After fixation in 4% formaldehyde and processing in 70% ethanol, specimens harvested on the 3rd, 14th and 28th days were paraffin embedded; 3-*μ*m sections were taken throughout the graft, excluding regions immediately adjacent to the anastomotic cuffs. Sections were stained with hematoxylin and Masson kit (Shanghai Gefan Biological, China.), and staining sections were observed by a Leica-DM2000 biological microscope (Leica, Hefei, China). At least five equally spaced sections of the bypass grafts were analyzed for each specimen. Standard morphometric measurements were recorded, including intimal thickness, medial thickness, and adventitial thickness. These data were then calculated by ImageJ (National Institutes of Health, Bethesda, Md).

### Immunohistochemistry analysis

Paraffin-embedded tissues (3, 14 and 28-day grafts) were processed, and 3-*μ*m sections were taken throughout the graft, excluding regions immediately adjacent to the anastomotic cuffs. After deparaffinization, the antigen was recovered with a citrate buffer for 20 min at high temperature and pressure, then deactivated with a 3% H_2_O_2_ endogenous peroxidase for 20 min and block endogenous peroxidase with 5% bovine serum albumin for 10 min. Sections were treated overnight at 4°C with anti-PCNA monoclonal antibody (D3H8P; Cell Signaling), anti-VCAM1 antibody (ab134047; Abcam, 1:500 dilution), and anti-ICAM1 antibody (ab119871; Abcam, 1:200 dilution). Three separate sections per graft were analyzed for all markers. The slices were washed several times. Biotinylated secondary antibody working solution and horseradish peroxidase conjugated streptavidin working solution were added to the slices, respectively. The slices were washed in PBS (pH 7. 2) for 5 minutes. Diaminobenzidine tetrahydrochloride (DAB) was used to visualize and hematoxylin to counterstain. Lastly, the slices were dehydrated, and then mounted and sealed. All images were acquired by image signal acquisition and analysis system (Leica-DM2000, Hefei, China). PCNA index was used to describe the level of PCNA expression (PCNA index = the numbers of PCNA positive cells / the numbers of all cells). Average optical density was used to describe the standard of VCAM-1 and ICAM-1 expressions (average optical density = Integral optical density / area).

### Western blot assay of PCNA and VCAM-1 protein expression analysis

Total protein was isolated from grafted veins. After electrophoresis and electrotransfer, membranes were blocked with 5% skimmed milk and were incubated at 4°C with primary antibodies overnight (with anti-PCNA monoclonal antibody D3H8P; Cell Signaling, 1:1000 dilution; anti-VCAM1 antibody ab134047; Abcam, 1:3000 dilution; anti-β-Tubulin antibody GB11017; Servicebio, 1:2000 dilution). After being incubated with secondary antibody (goat anti-mouse IgG-HRP SE131; Solarbio, 1:3000 dilution), the membranes were washed with TBST. The membranes were then exposed on the PVDF film and an ECL coloring solution (PE0010, Solarbio) was added. Separated immunoreactive bands were processed by Odyssey v1.2 software. Gray values were measured based on the internal reference of β-Tubulin.

### Statistical analysis

Data were presented as mean ± standard deviation (SD) and were processed by SPSS v.21.0 software (Chicago, IL). Because data followed a normal distribution verified by the Kolmogorov–Smirnov test, comparisons among multiple groups were analyzed by one-way analysis of variance (ANOVA). Comparisons between two groups were analyzed by Fisher's least significant difference (LSD) test. A p-value < 0.05 was considered statistically significant.

## Results

The previously described operation method was used to establish the model of autologous jugular vein bypass in rats. After the procedure, the transplanted jugular veins were fully filled, and the blood vessels beat well. Local perivascular delivery of Pluronic-F 127 gel evenly covered the surface of the blood vessel. The incision was closed after the glue solidified. The incision healing and activity status of rats were checked every day after operation. Doppler ultrasound was performed on the day of euthanasia, which showed that the transplanted jugular vein blood did not clearly occlude. In the 28-day control group, the vein grafts were thickened and were slightly stiff, and the surrounding tissue adhesion was relatively obvious. Correspondingly, veins in the high-dose adiponectin group did not expand significantly, and the surrounding tissues were easy to separate (Fig 1).

**Figure 1:** Intraoperative and postoperative photographs and Doppler ultrasound photographs. (A) a. Intraoperative photographs of control and high-dose adiponectin groups; (B) postoperative photograph in the fourth week of control and high-dose adiponectin groups; (C) Doppler ultrasound photographs on the day of euthanasia in the control and high-dose adiponectin groups.

### Adiponectin attenuates vein graft intimal, medial, and adventitial hyperplasia

Morphometric analysis of grafts explanted after bypass (Figure 2A) showed decreased intimal, medial, and adventitial thickness after perivascular delivery of adiponectin. Quantification of the intimal, medial, and adventitial thickness was performed by at least five equally spaced sections along the nonanastomotic vein for each bypass graft. In view of the three time points of day 3, day 14, and day 28, there was no significant difference in the thickness of the intima, media, and adventitia between the control group and the vehicle gel group, which meant the application of Pluronic F-127 gel as the load gel will not affect the pathophysiology of vein grafts. To be specific, the intimal thickness of the vein graft in the H-ADP group diminished significantly on day 14 and day 28 compared with the control group and the vehicle gel group. Similarly, intimal thickness in the L-ADP group evidently decreased more than that in the control group and the vehicle gel group on day 14 and day 28 (Figure 2B, Table 1).

**Figure 2:** Local perivascular delivery of adiponectin attenuates vein graft intimal, medial, and adventitial hyperplasia within rat vein grafts. (A) Light micrographs of Masson-stained sections of grafted veins (200× magnification, bar: 100*μ*m). (B) The change of neointimal thickness; (C) The change of medial thickness; (D) The change of adventitial thickness; (E) intima-to-media (I/M) ratio. Results are presented as mean ± SD. *P < 0.05 versus control group, **P < 0.01 versus control group. #P < 0.05 versus vehicle gel group, ##P < 0.01 versus vehicle gel group. I: intima; M: media.

**Table 1:** The thickness of intima, media, and adventitia in the control, vehicle gel (pluronic-F127 gel), L-ADP (low-ADP dose), and H-ADP (high-ADP dose) groups

Groups	Time(day)	Control N=6	Vehicle gel N=6	L-ADP N=6	H-ADP N=6	Analysis
Intima thickness (μm)	3	25.47±1.02	25.10±1.53	26.55±1.05	26.46±0.71	ANOVA
	14	111.69±5.31	106.62±5.57	75.12±4.94** ##	75.46±3.59** ##	ANOVA
	28	196.09±6.78	190.44±7.68	128.30±6.01** ##	96.96±5.45** ##	ANOVA
Media thickness (μm)	3	94.00±4.19	95.47±3.38	76.93±5.30** ##	69.03±5.94** ##	ANOVA
	14	107.19±3.66	107.41±5.48	93.86±3.17** ##	82.83±4.13** ##	ANOVA
	28	158.83±8.39	157.75±8.75	109.67±7.16** ##	104.02±7.12** ##	ANOVA
Adventitia thickness (μm)	3	56.09±3.42	53.03±4.59	54.51±3.95	59.21±4.28	ANOVA
	14	112.03±7.08	111.87±6.35	85.04±6.35** ##	86.52±5.20** ##	ANOVA
	28	152.31±3.55	154.49±6.55	95.70±6.05** ##	90.15±4.87** ##	ANOVA

The values of thickness are presented as mean ± SD (N=6).

* p < 0.05 versus control group,

** p < 0.01 versus control group.

# p < 0.05 versus vehicle gel group,

## p < 0.01 versus vehicle gel group.

Compared with intimal hyperplasia, the average hyperplasia in the grafted veins occurred earlier; the thickening of the average in the control and the vehicle gel group on day 3 was more obvious than that in the L-ADP and H-ADP groups. Similarly, the thickness of the medial tissue decreased in the high-dose adiponectin groups when compared with the control group and the vehicle gel group on day 14 and day 28. The same tendency also existed in the low-dose adiponectin group on day 14 and day 28 (Figure 2C, Table 1).

The intima-to-media ratios in the L-ADP and H-ADP groups were higher when compared to the control and vehicle gel groups on day 3. After, the intima-to-media ratio was notably lower in the H-ADP group on day 14 and day 28 when compared to the control and vehicle gel groups. Data from the L-ADP group showed a similar tendency at day 14 and day 28 when compared to the control and vehicle gel groups (Figure 2E).

In addition, our study found that the application of adiponectin can also inhibit the adventitia hyperplasia of grafted veins in both of the different dose groups. Adventitia thickness was attenuated by high-dose adiponectin when compared to the control and vehicle gel groups on day 3, day 14, and day 28. The same trend occurred in the low-dose adiponectin treatment groups when compared to the control and vehicle gel groups on day 3, day 14, and day 28 (Figure 2D, Table 1).

### Adiponectin decreases cell proliferation after vein grafting

Cell proliferation was quantified by the PCNA index of intima and adventitia (total number of PCNA-positive cells divided by the total number of nucleated cells) at 3 time-points after bypass (Figure 3A, 3B). The cell proliferation between the control and the vehicle group in both intima and adventitia was similar at 3 points of time. High-dose adiponectin-treated veins showed minimal proliferation in intima (26%) and adventitia (10%) three days after bypass as compared to the control and vehicle gel groups. Compared to the control (intima: 36%; adventitia: 36%) and vehicle gel (intima: 37%; adventitia: 37%) groups, proliferation diminished in the L-ADP group both in intima (29%) and adventitia (19%) on the third day. Moreover, proliferation suppression of the H-ADP group was also more obvious in adventitia than that of the L-ADP group on day 3. Proliferation was pronounced in vein grafts on day 14 (intima, 48%; adventitia, 46%) and day 28 (intima, 61%; adventitia, 51%) in the control group, but also on day 14 (intima, 47%; adventitia, 46%) and day 28 (intima, 59%; adventitia, 50%) in the vehicle gel group. Perivascular application of high-dose adiponectin exhibited 34% proliferation in intima and 20% in adventitia on day 14 when compared to the control and vehicle gel groups. Proliferation in the L-ADP group was visibly lower in both intima (43%) and adventitia (37%) on day 14 when compared to the control and vehicle gel groups. The differences can also be found in intima and adventitia between two different dose groups on day 14. Proliferation inhibition was also evident on day 28 in the L-ADP group, 49% proliferation of intima and 43% of adventitia, which illustrated significant differences when compared to the control and the vehicle gel groups. The expression of PCNA in the H-ADP group showed a similar trend in intima (38%) and adventitia (29%) on day 28. Comparison between two different adiponectin groups also showed notable differences in intima and adventitia on day 28 (Figure 3C, 3D). The expression of PCNA determined by Western blot also demonstrated the same effects of adiponectin (Figure 4A, 4B).

**Figure 3:** Adiponectin decreases cell proliferation. (A) PCNA index of intima determined by immunohistochemistry (200×magnification); (B) PCNA index of adventitia determined by immunohistochemistry (200×magnification); (C) PCNA index of intima; (D) PCNA index of adventitia. Data represent mean ± SD. *p < 0.05 versus control group, **p < 0.01 versus control group. #p < 0.05 versus vehicle gel group, ##p < 0.01 versus vehicle gel group.

**Figure 4:** Inhibition of PCNA and VCAM-1 expressions by delivery of adiponectin. (A) Expressions of PCNA and VCAM-1 in all groups at three points in time after operation; (B) Expression of PCNA determined by Western blot; (C) Expression of VCAM-1 determined by Western blot. Data represent mean ± SD. *p < 0.05 versus control group, **p < 0.01 versus control group. #p < 0.05 versus vehicle gel group, ##p < 0.01 versus vehicle gel group.

### Adiponectin decreases VCAM-1 and ICAM-1 expressions after vein grafting

VCAM-1 was expressed in the early stages, but there was no difference in each group on day 3. Difference was not observed between the control and the vehicle gel group. The expression of VCAM-1 in the high-dose adiponectin treatment group was significantly suppressed in relation to the control and vehicle gel groups on day 14 (0.25 versus 0.31, 0.30) and day 28 (0.30 versus 0.50, 0.50). The expression of VCAM-1 in the L-ADP group was also inhibited in relation to the control and vehicle gel groups on day 14 (0.26) and day 28 (0.40). *In addition*, the difference between the H-ADP and the L-ADP groups was also marked on day 28 (Figure 5A, Figure 5C). Results determined by Western blot also illustrated a similar tendency (Figure 4A, Figure 4C).

**Figure 5:** Adiponectin decreases cytokine expressions after vein grafting. (A) VCAM-1 expression determined by immunohistochemistry (200×magnification); (B) ICAM-1 expression determined by immunohistochemistry (200×magnification); (C) VCAM-1expression; (D) ICAM-1 expression. Data represent mean ± SD. *p < 0.05 versus control group, **p < 0.01 versus control group. #p < 0.05 versus vehicle gel group, ##p < 0.01 versus vehicle gel group.

ICAM-1 expression analyzed by ImageJ was rarely observed in the early stages. The expressions of ICAM-1 showed no prominent difference in the H-ADP group (0.10 versus 0.18 in control group, 0.19 in vehicle gel group) and the L-ADP group (0.10 versus control and vehicle gel group) until day 28 (Figure 5B). No difference could be observed between the two different dose adiponectin groups at the three point in time.

## Discussion

Despite advances in CAD therapies, CABG remains the main surgical treatment, and thus restenosis continues to plague the patency of vein grafts.^26^ Many experiments have tried to better understand the mechanisms of venous-graft failure and to identify new approaches to reduce vessel injury and to improve healing.^27–29^ Our study demonstrated that adiponectin delivered to the external surface of rat vein grafts at the time of implantation through Pluronic-F gel not only reduced intimal hyperplasia on day 28 compared with bypass-only controls, but can also decrease medial and adventitial hyperplasia. Our results suggest that reduced anti-proliferative activity and cytokine expression (VCAM-1, ICAM-1) in the graft wall are likely to be involved in this effect. These results demonstrate the role of ADP in preventing the vein graft restenosis and highlight the potential mechanism.

The complicated remodeling process of vessels results in restenosis of the vein grafts, but the specific mechanism is still unclear. Studies have shown that the process of venous-graft restenosis includes thrombosis, intimal hyperplasia, neointimal formation, and atherosclerosis.^3^ After vessel transplantation, vascular smooth muscle cell proliferation and migration are affected by changes in endothelial cell dysfunction, inflammation, hemodynamics, among other factors.^11^ In addition, the adventitia of the blood vessel is also damaged. The activated adventitial fibroblasts transform into myofibroblasts, and then proliferate and migrate to the media and intima, where they synthesize and release a variety of cytokines to promote smooth muscle cell proliferation and migration.^30–32^ Studies have revealed that adiponectin can be bound to adiponectin receptors expressed in adventitial fibroblasts and play a key role. Furthermore, many studies have reported that adiponectin has anti-inflammatory and anti-atherosclerotic effects on the cardiovascular system.^33^ However, the effects of ADP on preventing restenosis remain largely unknown. The present study primarily demonstrated the potential efficacy of ADP in inhibiting vein graft hyperplasia.

In this study, adiponectin was applied to the rat autologous vein graft model to stimulate the pathophysiological process of vein grafts after CABG. The thickness of intima in the adiponectin groups was found to be significantly lower than that in the bypass-only group and vehicle-gel treatment group, indicating that adiponectin has an effect on inhibiting intima hyperplasia. Moreover, the higher-dose adiponectin was more effective in this study. Interestingly, we found that the PCNA index of intima and adventitia decreased in the adiponectin treated groups as well, demonstrating that adiponectin may well have an inhibitory effect on cell proliferation in the intima and adventitia of grafted veins. This study provides only limited insight into the mechanisms through which ADP reduced vein graft intima in this model. However, comparing our data with that taken in the context of previous studies, it can be concluded that adiponectin was not only able to inhibit intimal hyperplasia, but also to inhibit the proliferation of myofibroblasts and limit its migration to the intima and media.^34,35^

However, the specific mechanism of the interaction between adiponectin and receptors still needs further study. The pharmacokinetics of perivascular delivery of adiponectin also need to be better defined in future studies. Researchers have also shown that thrombosis plays a key role in the early phase of vein graft failure. The vein is subjected to a period of ischemia-reperfusion injury-leading to a reduction in endothelial nitric oxide synthase (eNOS) expressing, damage to endothelial cells and smooth muscle cells (SMCs), and the release of various prothrombotic mediators. Furthermore, the remodeling processes of the vein graft start within days after harvesting and grafting, leading to the formation of intimal hyperplasia.^25^ We believe ADP can exert an impact in both the early and intermediate stages. Our study only brings information about the short-term effect. Further research is warranted in order to understand the specific mechanism in the short-term and to know how ADP influences the long-term outcome.

After the endothelium is damaged, an early pathophysiological process of restenosis, the adhesion molecules ICAM-1 and VCAM-1 are released, which lead to adhesion and infiltration of monocytes / macrophages that differentiate and engulf a large amount of ox-LDL, and then form foam cells. In our experiments, we detected the expression of VCAM-1 and ICAM-1 by immunohistochemistry and found that the values of VCAM-1 and ICAM-1 in the adiponectin group were markedly lower than those in the vehicle-gel treatment group and the bypass-only group, which elucidated how the application of adiponectin can downregulate the expression of VCAM-1 and ICAM-1 to promote dysfunction of endothelium.

## Conclusion

The present study confirmed that the local perivascular delivery of adiponectin attenuates vein graft hyperplasia in a rat carotid bypass model. This effect appears to be mediated by both decreased cell proliferation and a downregulated expression of VCAM-1 and ICAM-1 within the graft. Although the short-term effects of adiponectin seem promising, the long-term effects and clinical significance of adiponectin in CABG need to be studied in the future. We hope that these studies will eventually be translated into clinical application in the prevention of restenosis and bypass graft failure.
